# Precipitation and soil moisture data in two engineered urban green infrastructure facilities in New York City

**DOI:** 10.1016/j.dib.2020.106225

**Published:** 2020-08-25

**Authors:** Bita Alizadehtazi, Franco A. Montalto

**Affiliations:** Department of Civil, Architectural & Environmental Engineering, Drexel University, 3141 Chestnut Street, Philadelphia, PA 19104, USA

**Keywords:** Urban soil moisture, Green infrastructure, Ecohydrology, Hydraulic loading ratio, Bioretention, Stormwater, Precipitation

## Abstract

This paper archives spatiotemporal volumetric moisture content (VMC) and associated precipitation datasets collected between 2012 and 2014 at different depths in two different New York City green infrastructure (GI) (e.g. bioretention) facilities, termed Site 1 and Site 2, respectively. The two sites are similar in both design and monitoring set up, and are located within two kilometers of one another, but differ in terms of hydraulic loading ratio (HLR). Both sites were designed and instrumented specifically to facilitate a comparison of the hydrologic fluxes within the two GI facilities. Site 1 receives only direct rainfall and is hydrologically isolated from the surrounding impervious surfaces (HLR = 1); Site 2 receives both direct precipitation and street runoff through a curb cut inlet (HLR = 3.8). Monitoring was conducted both inside (L plots) and outside (G plots) weighing lysimeters that were installed at both sites and planted with similar vegetation. Each L and G plot was equipped with five soil moisture sensors installed at 5, 10, 20, 30, and 50 cm depths in a circular pattern. This dataset is associated with the original research presented in “Observed Variability in Soil Moisture in Engineered Urban Green Infrastructure Systems and Linkages to Ecosystem Services [1].”

## Specifications Table

**Table:** 

Subject	Civil and Structural Engineering
Specific subject area	Urban hydrology, Green infrastructure, Soil moisture, Remote sensing, Urban ecohydrology, Stormwater
Type of data	Tables, figures The data are stored in *.csv files in a zip package
How data were acquired	Precipitation and soil moisture were continuously monitored using Texas Electronics tipping bucket rain gauges and Decagon 5TE soil moisture sensors, respectively
Data format	Raw data
Parameters for data collection	Onsite precipitation at each site was monitored with two side-by-side Texas Electronics tipping bucket rain gages mounted at a height of four meters. Each L and G plot was equipped with five soil moisture sensors installed in a circular pattern at 5, 10, 20, 30, and 50 cm depths.
Description of data collection	Precipitation (mm) and soil moisture (m^3^/m^3^) data were logged on a Campbell Scientific CR1000 data logger at 5-min time intervals and transmitted to a server via a cell modem connection
Data source location	Site 1 is a bioretention facility located within the Colfax Street and Murdock Avenue Greenstreet, Queens, NY, USA (40.702, −73.743) Site 2 is a bioretention facility located within the 116th Avenue and Nashville Boulevard Greenstreet, Queens, NY, USA (40.698, −73.744) Both sites are the property of the New York City Department of Parks and Recreation (New York, NY USA). The data is warehoused at the Sustainable Water Resource Engineering Lab at Drexel University (Philadelphia, PA, USA)
Data accessibility	With the article
Related research article	B. Alizadehtazi, P.L. Gurian, F.A. Montalto, Observed Variability in Soil Moisture in Engineered Urban Green Infrastructure Systems and Linkages to Ecosystem Services, Journal of Hydrology. 590 (2020) 125381.

## Value of the Data

• Green infrastructure facilities are being engineered throughout the world to manage urban stormwater but continuous monitoring of the hydrologic fluxes through these decentralized facilities is still uncommon.
• The manuscript contains difficult to obtain soil moisture and precipitation data collected in two green infrastructure facilities over a multi-year observation period.
• Continuous monitoring of in-situ soil moisture can be logistically challenging and costly both in terms of time and resources.
• The data is useful to researchers who are interested in the ecosystem services provided by urban green infrastructure facilities, especially as they relate to soil moisture.
• The data can be used to develop and to calibrate certain types of urban hydrologic and hydraulic models as well as ground-truth other spatial data sets (e.g. soil moisture and/or precipitation from satellites).
• Green infrastructure is a billion-dollar industry within the United States, and growing as well in other countries. Urban stormwater utilities need performance data in order to justify and potentially expand/adapt this investment.

## 1 Data Description

**Fig. 1:**
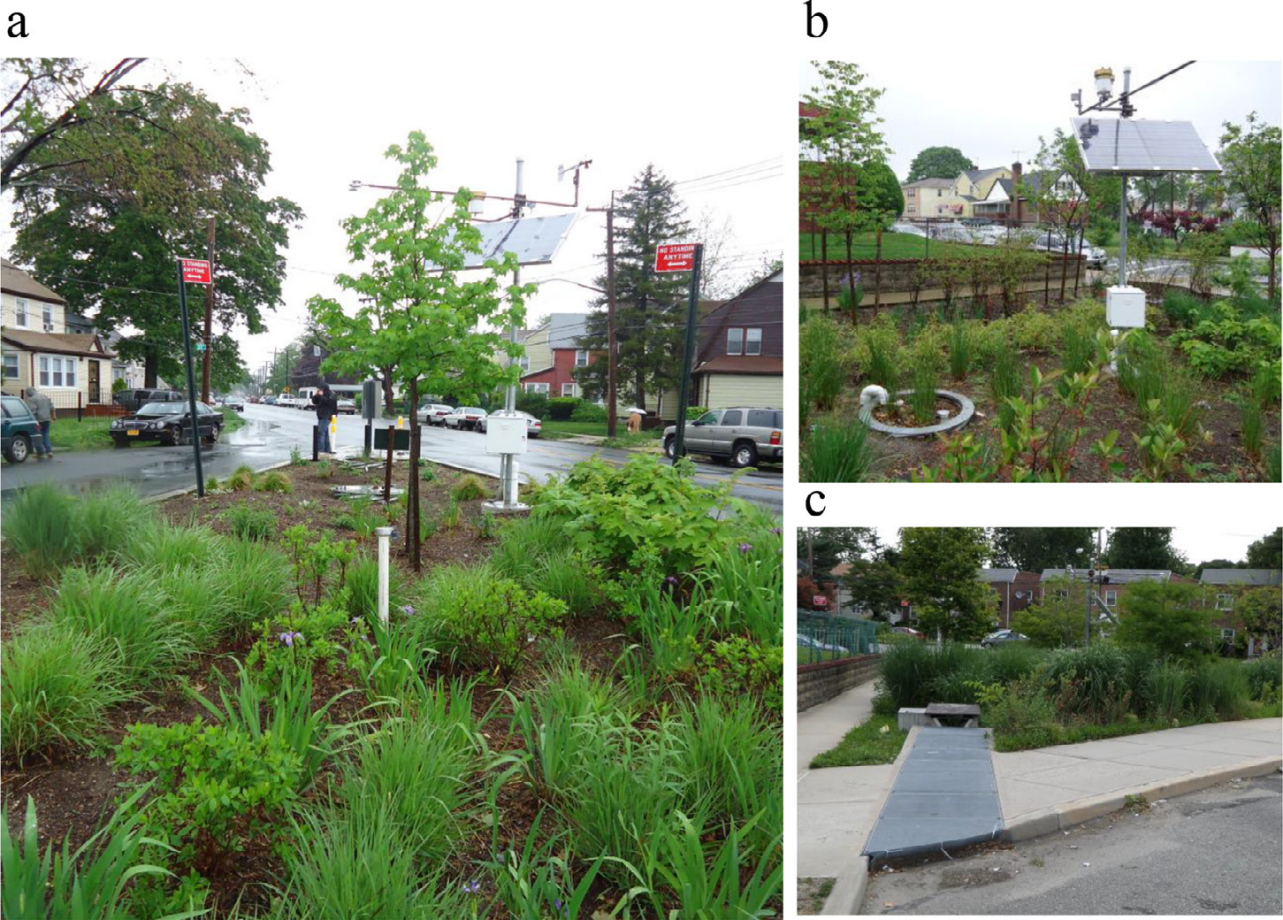
Bioretention facilities: a) picture of Colfax and b and c) pictures of Nashville.

**Fig. 2:**
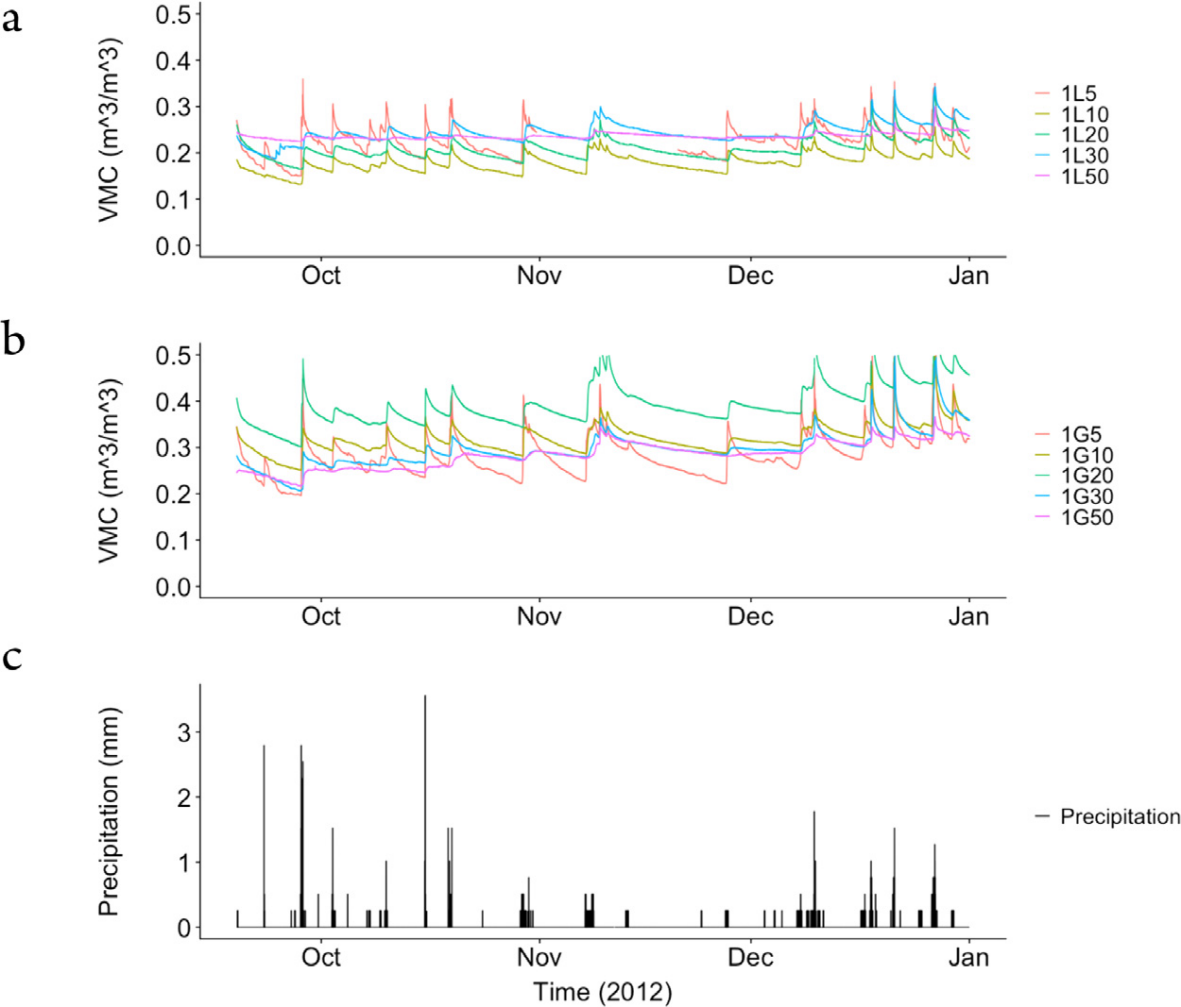
Observed volumetric moisture content (VMC) at 5, 10, 20, 30 and 50 cm depths and precipitation for: a) Site 1 inside the lysimeter plot (L), b) Site 1 outside the lysimeter plot (G), and c) the simultaneous precipitation in millimeters per each 5-min sampling period.

**Fig. 3:**
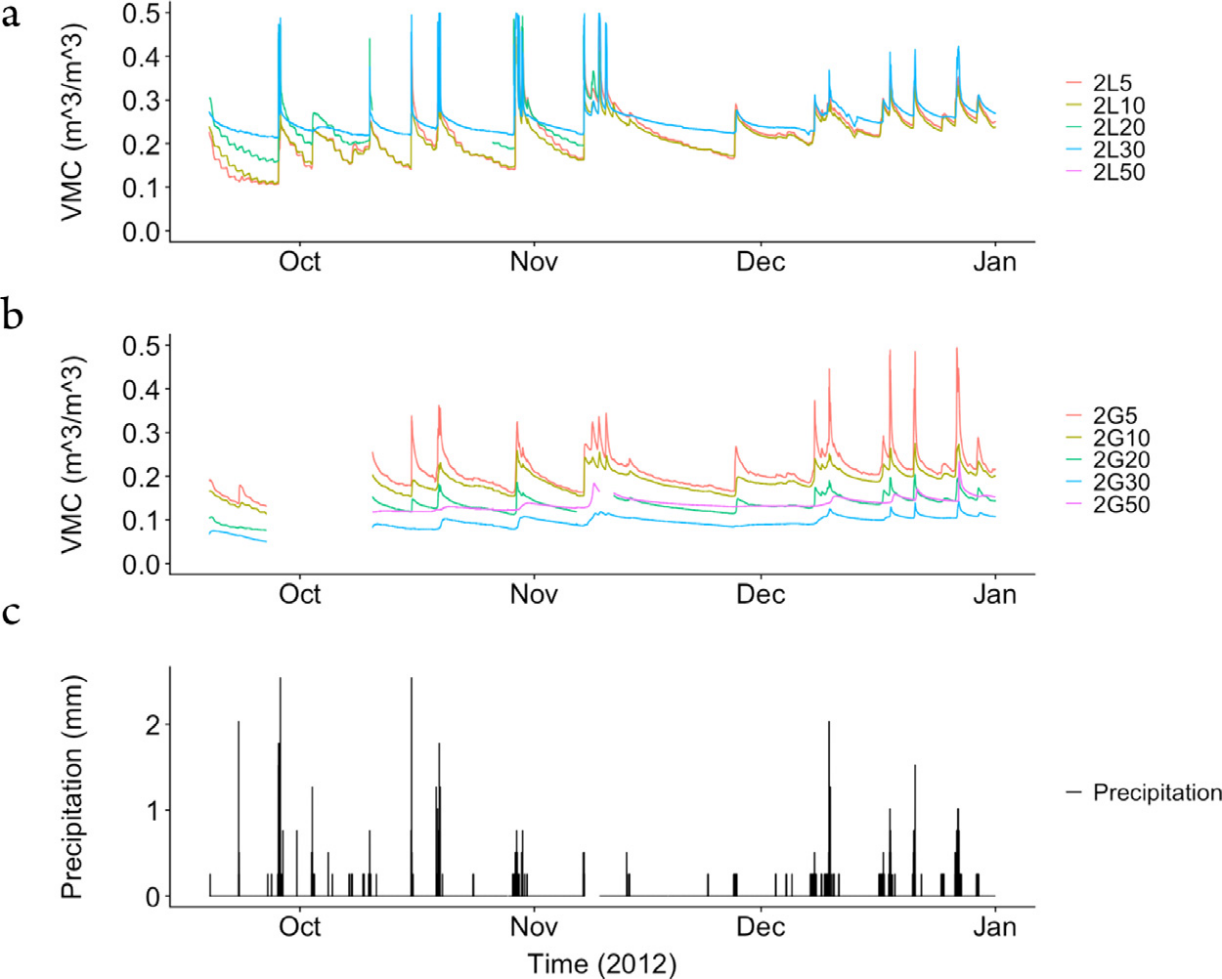
Observed volumetric moisture content (VMC) at 5, 10, 20, 30 and 50 cm depths and precipitation for: a) Site 2 inside the lysimeter plot (L), b) Site 2 outside the lysimeter plot (G), and c) the simultaneous precipitation in millimeters per each 5-min sampling period.

**Fig. 4:**
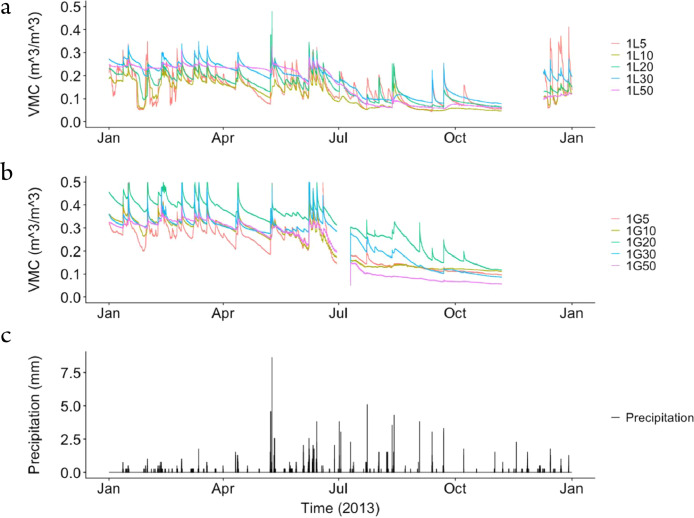
Observed volumetric moisture content (VMC) at 5, 10, 20, 30 and 50 cm depths and precipitation for: a) Site 1 inside the lysimeter plot (L), b) Site 1 outside the lysimeter plot (G), and c) the simultaneous precipitation in millimeters per each 5-min sampling period.

**Fig. 5:**
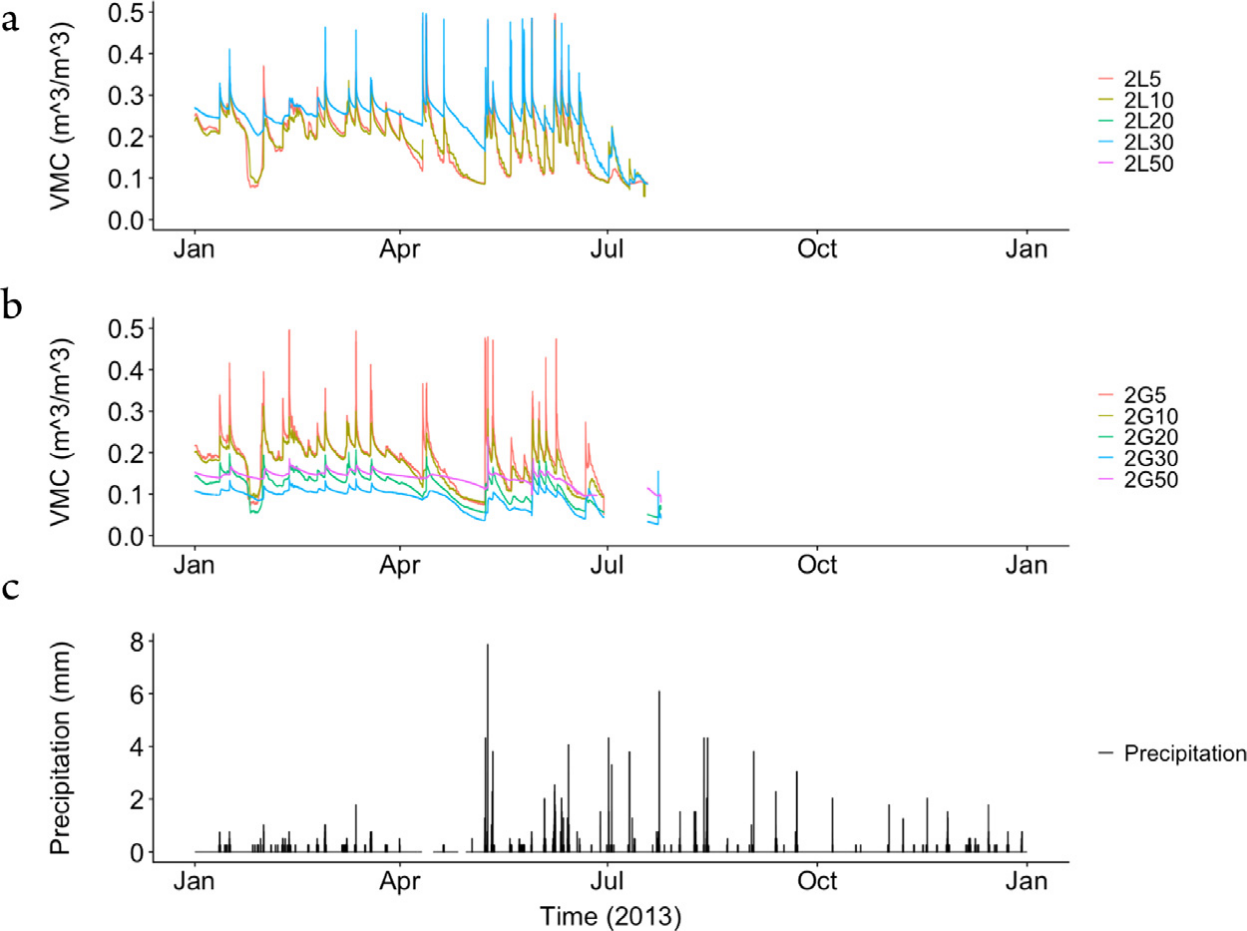
Observed volumetric moisture content (VMC) at 5, 10, 20, 30 and 50 cm depths and precipitation for: a) Site 2 inside the lysimeter plot (L), b) Site 2 outside the lysimeter plot (G), and c) the simultaneous precipitation in millimeters per each 5-min sampling period.

**Fig. 6:**
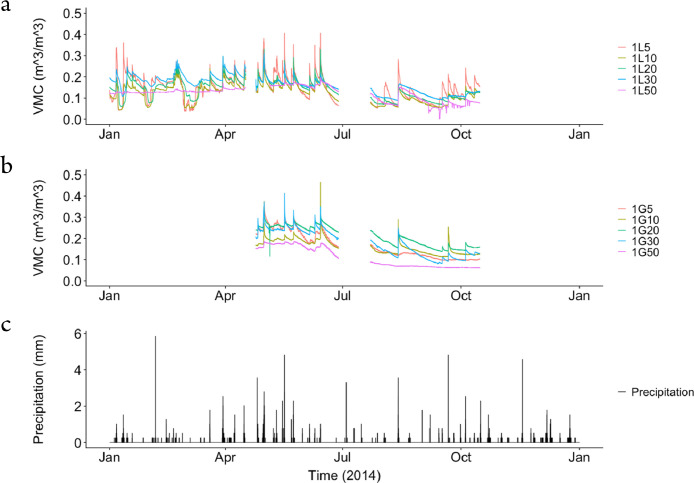
Observed volumetric moisture content (VMC) at 5, 10, 20, 30 and 50 cm depths and precipitation for: a) Site 1 inside the lysimeter plot (L), b) Site 1 outside the lysimeter plot (G), and c) the simultaneous precipitation in millimeters per each 5-min sampling period.

**Fig. 7:**
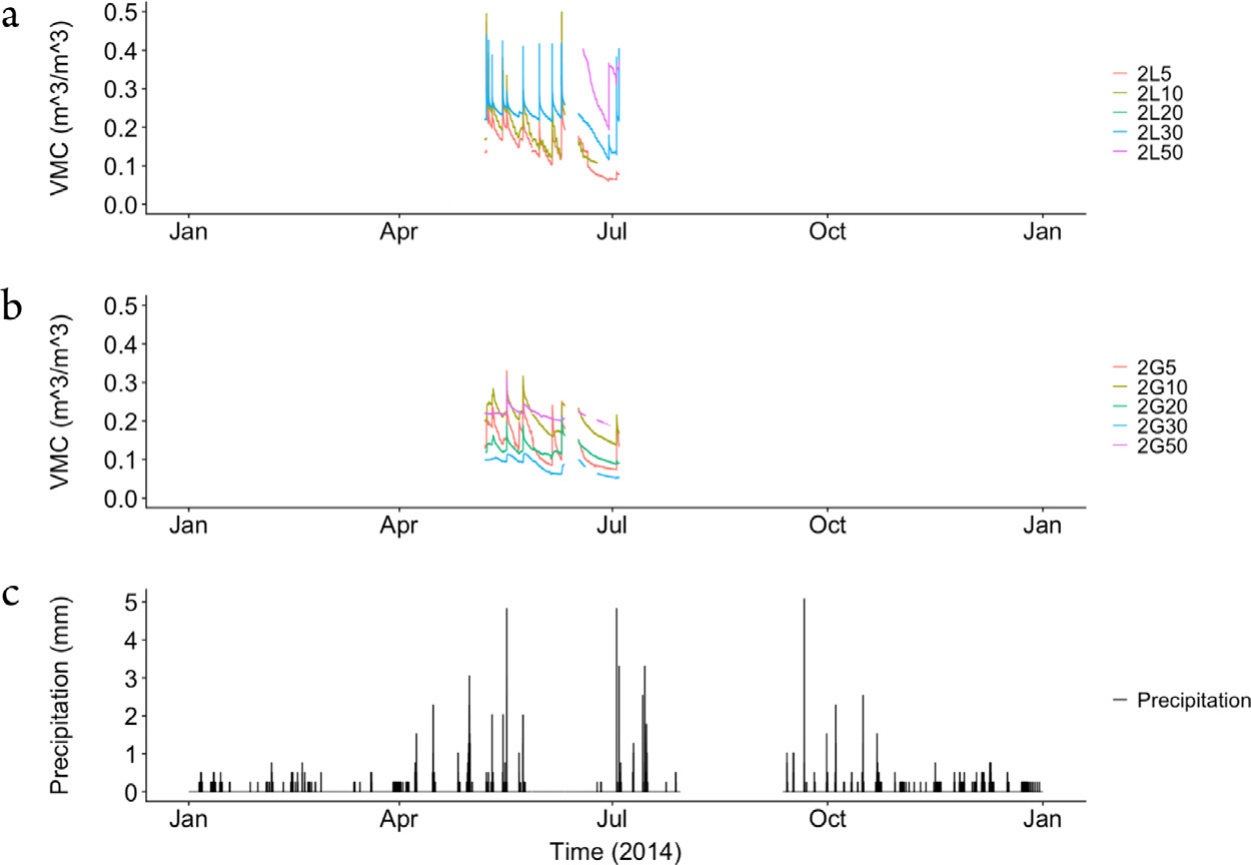
Observed volumetric moisture content (VMC) at 5, 10, 20, 30 and 50 cm depths and precipitation for: a) Site 2 inside the lysimeter plot (L), b) Site 2 outside the lysimeter plot (G), and c) the simultaneous precipitation in millimeters per each 5-min sampling period.

The dataset contains raw data collected at two different bioretention facilities (Fig. 1). The raw precipitation and soil moisture data are stored in two files (Site1.csv and Site2.csv). Inside the files, data labels were developed indicating the facility site name, plot location, and depth, at which soil moisture measurements were made. The nomenclature used to refer to each soil moisture sensor is a concatenation of the site number (1,2), the plot location (L or G), and the soil sensor depth (5,10, 20, 30, 50). For example, the 1L5 refers to the lysimeter plot at Site 1, and specifically the soil sensor at 5 cm depth. The two rain gages at each site are referred to as Rainfall1 and Rainfall2, respectively for each site. Technical specifications of these sensors are provided in Table 1. The soil moisture time series data collected at both locations (L or G) at each of the two sites are presented in Fig. 2, Fig. 3, Fig. 4, Fig. 5, Fig. 6, Fig. 7, for 2012 (Figs. 2 and 3), 2013 (Fig. 4, Fig. 5), and 2014 (Fig. 6, Fig. 7). To optimize visualization of the data, VMC values up to 0.5 m^3^/m^3^ are shown in the figures. The accompanying *.csv files include all the raw data values (including those that exceed the maximum value of vertical axis used in these plots).

**Table 1:** Technical specifications of the equipment and sensors.

Measured parameter	Equipmentmanufacture/model	Specifications	Installationheight/ depth
Logger	Campbell Scientific, Inc. CR1000	Logged at 5 min intervals	__
Precipitation	Texas Electronics, Inc. Series 525 Rainfall Sensor	Up to 50 mm/h: ± 1%	4 m height
Soil moisture	Decagon Devices 5TE Soil Sensor	Soil moisture: ± 1–3% VWC	5 cm 10 cm 20 cm 30 cm 50 cm

## 2 Experimental design, materials and methods

Soil moisture and precipitation data were collected in two bioretention facilities located within two kilometers of one another in Queens, New York City (NYC). The two NYC sites were profiled in 2018 as examples of nature-based solutions to stormwater in WWAP [2].

Site 1 (Fig. 1a) is a bioretention facility located within the Colfax Street and Murdock Avenue Greenstreet, Queens, NY, USA (40.702, −73.743) built in 2010–11 for research purposes. This site receives only direct rainfall and is hydrologically isolated from surrounding impervious surfaces (HLR = 1). Site 2 (Fig. 1b and c) is a bioretention facility located within the 116th Avenue and Nashville Boulevard Greenstreet, Queens, NY, USA (40.698, −73.744). Site 2, also built in 2010–11, receives both direct precipitation and street runoff through a curb cut inlet (HLR = 3.8). Both sites were designed with similar vertical soil profiles, consisting of 60 cm of loamy sand on top of a thinner but variable depth layer of crushed stone. The native soils underlying the facility were sandy and thus did not hinder infiltration.

This paper archives data gathered from 2012 to 2014 using rain gauges and soil moisture sensors installed at both sites. Onsite precipitation was monitored with two, side-by-side Texas Electronics tipping bucket rain gages mounted at a height of four meters. The rain gages have a 0.2 m orifice and 0.254 mm resolution per tip. At each site, soil moisture monitoring was conducted at two locations, inside and outside weighing lysimeters, termed “L” and “G” plots, respectively. All locations were planted with similar vegetation. Each L and G plot was equipped with five soil moisture sensors installed at 5, 10, 20, 30, and 50 cm depths in a circular pattern to avoid electrical interference between them. The soil sensors were calibrated to the specific soil type. Data collected at each site was logged on a Campbell Scientific CR1000 data logger at 5-min time intervals and transmitted via a cell modem to a server for real time viewing.

The lysimeter at Site 1 receives only direct precipitation, like the rest of the site. To ensure that the lysimeter at Site 2 is dosed with runoff at the same HLR as the rest of its site, a specially designed flow diversion box and orifice was installed at the inlet. The flow diversion box directs runoff to the lysimeter between mid-April and mid-October only. During the colder winter months, the Site 2 lysimeter receives direct rainfall only, to avoid pipe rupture due to water expansion during freezing conditions.

## Declaration of Competing Interest

The authors declare that they have no known competing financial interests or personal relationships which have, or could be perceived to have, influenced the work reported in this article.

## Appendix Supplementary materials

Image, application 1
